# In Vivo Study of Transverse Carpal Ligament Stiffness Using Acoustic Radiation Force Impulse (ARFI) Imaging

**DOI:** 10.1371/journal.pone.0068569

**Published:** 2013-07-05

**Authors:** Zhilei Liu Shen, D. Geoffrey Vince, Zong-Ming Li

**Affiliations:** 1 Department of Biomedical Engineering, Cleveland Clinic, Cleveland, Ohio, United States of America; 2 Department of Orthopaedic Surgery, Cleveland Clinic, Cleveland, Ohio, United States of America; 3 Department of Physical Medicine and Rehabilitation, Cleveland Clinic, Cleveland, Ohio, United States of America; University of Pittsburgh, United States of America

## Abstract

The transverse carpal ligament (TCL) forms the volar boundary of the carpal tunnel and may provide mechanical constraint to the median nerve, leading to carpal tunnel syndrome. Therefore, the mechanical properties of the TCL are essential to better understand the etiology of carpal tunnel syndrome. The purpose of this study was to investigate the in vivo TCL stiffness using acoustic radiation force impulse (ARFI) imaging. The shear wave velocity (SWV) of the TCL was measured using Virtual Touch IQ^TM^ software in 15 healthy, male subjects. The skin and the thenar muscles were also examined as reference tissues. In addition, the effects of measurement location and ultrasound transducer compression on the SWV were studied. The SWV of the TCL was dependent on the tissue location, with greater SWV values within the muscle-attached region than those outside of the muscle-attached region. The SWV of the TCL was significantly smaller without compression (5.21 ± 1.08 m/s) than with compression (6.62 ± 1.18 m/s). The SWV measurements of the skin and the thenar muscles were also affected by transducer compression, but to different extents than the SWV of the TCL. Therefore to standardize the ARFI imaging procedure, it is recommended that a layer of ultrasound gel be maintained to minimize the effects of tissue compression. This study demonstrated the feasibility of ARFI imaging for assessing the stiffness characteristics of the TCL in vivo, which has the potential to identify pathomechanical changes of the tissue.

## Introduction

The transverse carpal ligament (TCL) forms the volar boundary of the carpal tunnel and plays a critical role in carpal tunnel mechanics. The TCL stabilizes the carpal bones in the transverse direction [1,2], serves as an anchor for the thenar and hypothenar muscles [3], and acts as a pulley for the flexor tendons [4]. During wrist flexion and finger motion, the flexor tendons and the median nerve migrate volarly and push against the TCL [5,6]. Therefore, the TCL can potentially compress the median nerve, causing carpal tunnel syndrome. The mechanical constraint imposed by the TCL to the median nerve is further illustrated by TCL transection as a standard surgical treatment for carpal tunnel syndrome. In addition, mechanical stimulation to the TCL during thenar muscle contraction may cause tissue remodeling and lead to thickening and stiffening of the TCL [7]. Therefore, investigating the mechanical properties of the TCL may lead to a better understanding of carpal tunnel syndrome etiology.

Numerous studies have investigated the mechanics of the TCL while examining the mechanics of the carpal tunnel [1,2,8–10]. For example, Garcia-Elias et al. [8] studied the role of the TCL in stabilizing the carpal bones while applying compressive forces in the dorso-volar direction. The stiffness of the carpal tunnel only decreased by 7.5% after the TCL was transected, suggesting that the TCL was not a major contributor to the carpal tunnel stability in the dorso-volar direction. Xiu et al. [2] applied inward and outward forces to the carpal bones in the transverse direction. The arch width change in response to the force application significantly increased after the TCL transection in the outward direction, indicating that the TCL played a mechanical role in stabilizing the carpal tunnel in the ulno-radial direction. Furthermore, the stiffness of exposed TCL in cadaveric hand specimens was investigated using an indentation testing machine [11]. The TCL stiffness was found to be 35.9 ± 3.5 N/mm when the wrist was in a neutral posture. Although the TCL has been examined as part of the carpal tunnel, the mechanical properties of an isolated TCL remain unclear.

Only a few mechanical studies have been performed on isolated TCLs directly. Using cadaveric bone-ligament-bone specimens, Garcia-Elias et al. [8] found that the TCL had an axial tensile stiffness of 131.8 ± 54 N/mm and a failure load of 343.6 ± 46 N. Holmes et al. [12] measured the mechanical properties of the TCL at different tissue locations using biaxial tensile testing. It was shown that the proximal segments of the TCL had greater elastic moduli than the distal ones and the radial segments had greater moduli than the ulnar ones. In addition, the largest elastic modulus of the TCL was found in the proximal radial region, with a mean of 2.8 MPa. Main et al. [13] investigated the compressive mechanical properties of the TCL in the volar/dorsal direction and reported that they were location-dependent, i.e. the stiffness values at the compression sites with muscle attachments were greater than those at the compression sites without muscle attachments. The preceding studies required invasive procedures to expose the TCL to investigate its mechanical properties. It has been technically challenging to study the in vivo mechanical properties of the TCL.

Acoustic radiation force impulse (ARFI) imaging is an ideal tool to assess the in vivo TCL stiffness because it is an ultrasound elastography technology capable of noninvasively measuring tissue stiffness. ARFI is achieved by mechanically exciting tissues with localized impulsive radiation force and resulting in shear wave propagation away from the region of excitation [14]. The shear wave velocity (SWV) is directly correlated with the tissue stiffness. In general, a greater SWV corresponds to a stiffer tissue. ARFI imaging has been used to study a variety of tissues, including liver [15], breast [16], kidney [17], spleen [18], prostate [19], pancreas [20], testes [21], thyroid [22], muscle and tendon [23]. The most notable application of ARFI is to detect liver fibrosis [24–26] as the fibrous tissues are usually stiffer than the surrounding tissues. Furthermore, some ARFI studies demonstrated the dependence of tissue stiffness on the amount of compression applied by the ultrasound transducer [23,27,28]. It was shown that the SWV measurements made with compression were larger than those made without compression for breast [27], kidney [28], muscle and tendon [23]. However, there is a lack of ARFI application to musculoskeletal tissues.

The purposes of this study were to investigate the in vivo TCL stiffness at different tissue locations by measuring the SWV using ARFI imaging and to examine the effects of compression from the ultrasound transducer on the SWV measurements. In addition to the TCL, the skin and the thenar muscles were examined by ARFI imaging as reference tissues to normalize the SWV of the TCL. We hypothesized that the SWV measurements of the TCL were dependent on the tissue location. We further hypothesized that the SWV measurements made without compression would be smaller than those made with compression.

## Materials and Methods

### Ethics Statement

Informed, written consent was obtained from each subject prior to the study. This study was approved by the Institutional Review Board at the Cleveland Clinic Foundation.

### Subjects

Fifteen healthy, male subjects were recruited for this study. The mean (standard derivation) age, height, and weight of the subjects were 30 (4) years, 1.76 (0.06) m, and 82 (13) kg, respectively. Exclusion criteria for subjects were self-reported musculoskeletal or neuromuscular disorders to the hand and wrist.

### Subject Positioning

**Figure 1:**
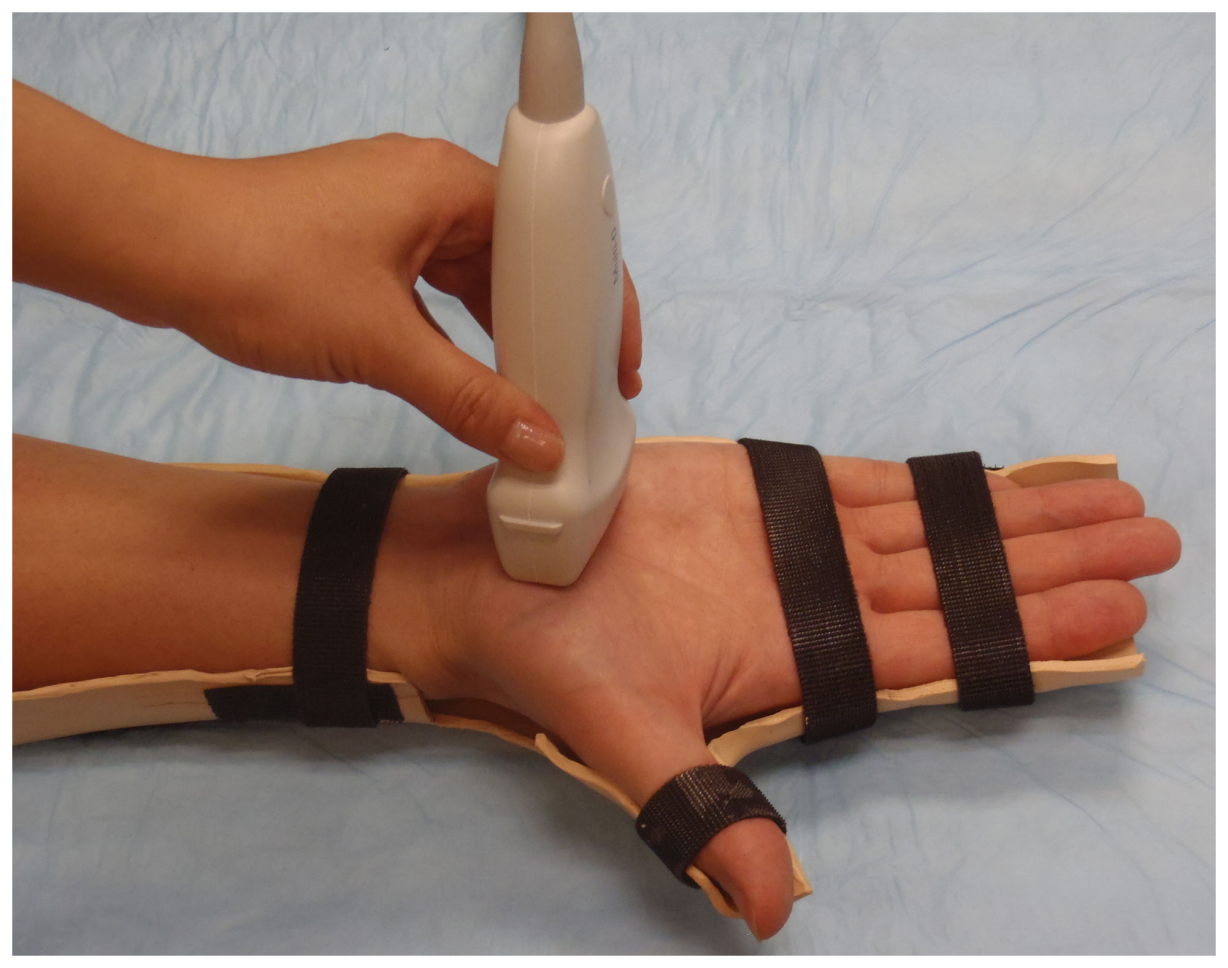
Positioning of the hand and ultrasound transducer for ARFI imaging. The right hand and forearm were stabilized in a custom-made splint using Velcro^®^ straps. The ultrasound transducer was placed at the distal level of the carpal tunnel.

Each subject sat in an upright position next to a testing table. The right hand and forearm were secured in a custom-made thermoplastic splint using Velcro^®^ straps (Figure 1). The hand was supinated with the wrist in an anatomically neutral position. The fingers were at full extension and the thumb was at 0° palmar abduction and 60° radial abduction. The digit positioning was maintained using Velcro^®^ straps.

### ARFI Imaging

A single operator conducted the ARFI imaging on all subjects. The operator was specifically trained for ultrasound imaging of the carpal tunnel. An ultrasound system (ACUSON S2000, Siemens Medical Solutions USA Inc., Mountain View, CA, USA) with a 9L4 linear array transducer was used for this study. Prototype ARFI imaging technology has been incorporated into this commercial ultrasound system in the form of Virtual Touch IQ^TM^ software. This software not only forms a two-dimensional color map of SWV, but also provides a quantitative assessment of tissue stiffness through SWV measurements. The SWV color map is generated by a pulse sequence consisting of up to 256 acquisition beam lines. For each beam line, sequencing of push pulses and tracking vectors is used to estimate the shear wave propagation time for each depth along the beam direction. The sequencing is then repeated for all the beam lines to form a SWV color map. The imaging frequency was set to 9 MHz and the image field depth was set to 3 cm. The transducer was placed at the distal level of the carpal tunnel. Its position was adjusted by the operator until the ridge of the trapezium and the hook of the hamate were clearly identified on the ultrasound monitor. In this orientation, the shear waves propagated along the transverse (ulno-radial) direction) of the TCL, resulting in SWV measurement in this direction.

Two sessions of ARFI imaging were performed on each subject to investigate the effects of compression from the transducer on the SWV measurements. During the first session (i.e. without compression), a visible ultrasound gel layer with a thickness of 2-8 mm was maintained between the transducer and the skin on the ultrasound monitor. The existence of a gel layer indicated a minimal compression from the transducer to the skin and the underlying tissues. This ultrasound imaging procedure was shown to improve ARFI imaging quality [29]. For the second session (i.e. with compression), a gentle force was applied by the transducer to the skin. The applied force was qualitatively controlled to diminish the skin fold of the palm (i.e. to flatten the curvature of the skin surface) while avoiding noticeable deformation in the deeper tissues such as blood vessels and muscles.

**Figure 2:**
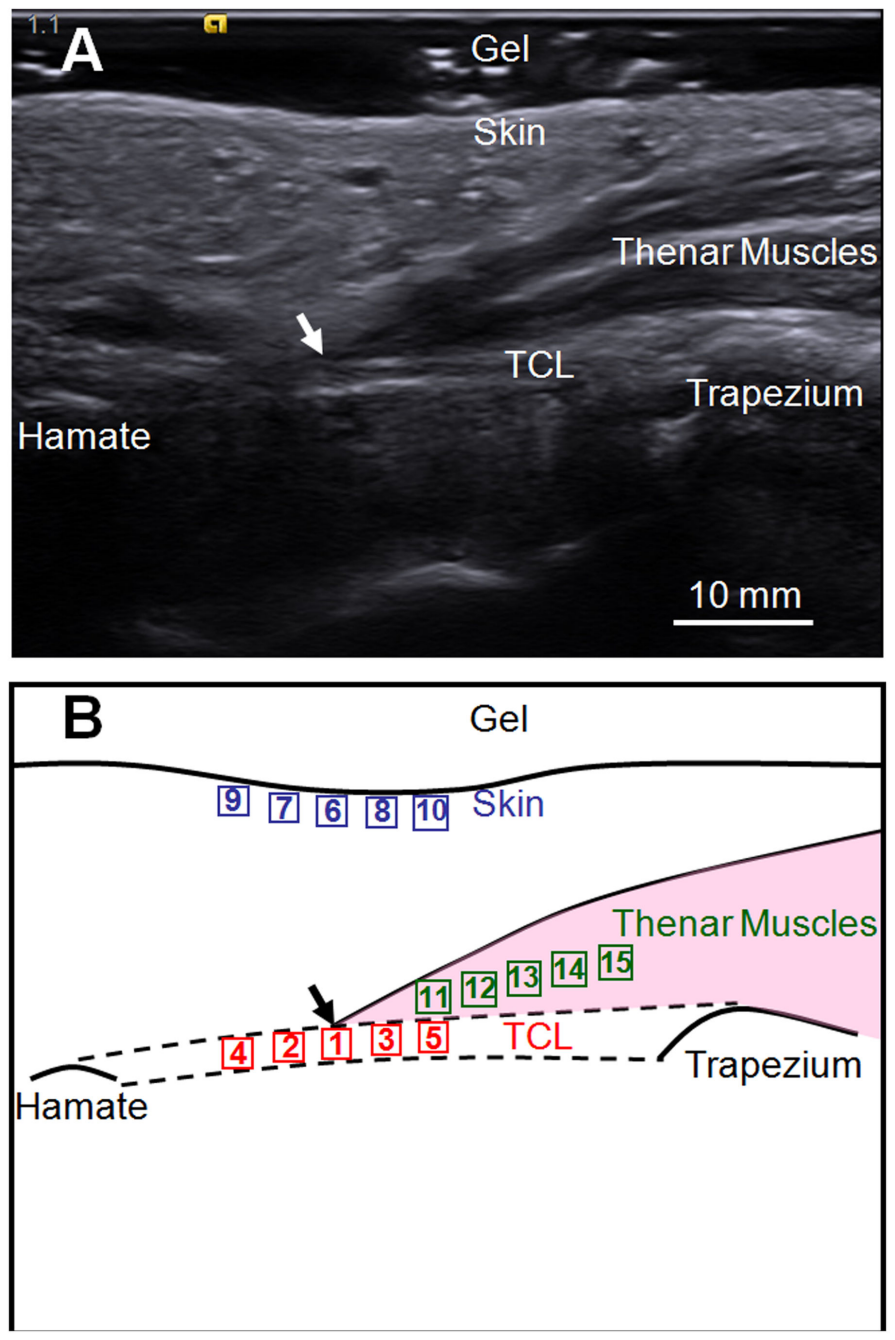
A representative ultrasound image and a respective schematic displaying selected regions of interest (ROIs). (A) Gray-scale B-mode ultrasound image where the transverse carpal ligament (TCL), the skin, the thenar muscles, the hamate, and the trapezium are reliably identifiable. The thenar muscle’s ulnar point (TUP) is indicated by an arrow. (B) A schematic to illustrate the fifteen ROIs that correspond to the shear wave velocity measurement locations, including five on the TCL (red), five on the skin (blue), and five on the thenar muscles (green). The sequence used for placing the ROI boxes is indicated by the number within each box. The TCL (dashed lines) is attached to the hamate and the trapezium. The thenar muscles are represented by the shaded area.

During each session, ten trials of ARFI imaging were performed using the Virtual Touch IQ^TM^ software and the ultrasound transducer was repositioned after each trial. During each trial, a conventional B-mode, gray-scale image was taken at the hook of the hamate level (Figure 2A). The upper and lower boundaries of the TCL were identified using the thenar muscles and the fascia surrounding the TCL as guides. Then, a SWV color map of 30 mm (width) × 20 mm (depth) was generated and overlaid on the gray-scale image. Care was taken to ensure that the color map covered the skin, the thenar muscles, the whole TCL, the hamate, the trapezium, and part of the carpal tunnel. The display scale of the color map was set to 0.5-10 m/s. Different colors represented different stiffness. In general, red, green, and blue areas on the color map corresponded to high, medium, and low stiffness regions, respectively.

Fifteen region-of-interest (ROI) boxes were manually placed within the color map to quantitatively measure the SWV, including five on the TCL, five on the skin, and five on the thenar muscles. Each ROI box had a preset size of 2 mm × 2 mm. The focus of this study was on the TCL, but the skin and the thenar muscles were also examined to investigate the effects of the compression on other soft tissues, and to explore whether they can be used as reference tissues to normalize the SWV of the TCL. The locations of these 15 ROI boxes are explained as follows and illustrated in Figure 2B. The first ROI box was placed on the TCL at the thenar muscles’ ulnar point (TUP), i.e. Location 1. The TUP was defined as the most ulnar aspect of the thenar muscles’ attachment to the TCL and was typically located in the middle of the TCL. The TUP was chosen because it was a unique anatomical feature that can be reliably identified on the ultrasound image as demonstrated in previous studies [7,30]. Two ROI boxes were then positioned next to the first box, corresponding to 2.7 mm ulnar to the TUP (Location 2) and 2.7 mm radial to the TUP (Location 3). Two more ROI boxes located on the TCL, corresponding to the 5.4 mm ulnar to the TUP (Location 4) and 5.4 mm radial to the TUP (Location 5). ROI boxes 6-10 were placed along the skin, with their x-coordinates matched to those of ROI boxes 1-5, respectively, and their y-coordinates at a depth of 1-1.5 mm below the skin surface. ROI boxes 11-15 were positioned along a line within the thenar muscles and were separated by ~1.8 mm. The SWV measurements corresponding to these ROI boxes were shown to the right of the ultrasound image. The maximum SWV value that the current Virtual Touch IQ^TM^ software with the 9L4 linear transducer can measure is 10 m/s. When the SWV is greater than 10 m/s, “HIGH” will be displayed instead of a numerical value. When the SWV cannot be determined due to poor shear wave signal to noise ratio, “NA” will be displayed.

### Statistical Analysis

A two-way (2×5) repeated measures analysis of variance (ANOVA) was used to investigate the effects of compression and measurement location on the SWV measurements of the TCL. The first independent variable, compression, had two levels: without compression and with compression. The second independent variable, location, had five levels: 5.4 mm ulnar to the TUP, 2.7 mm ulnar to the TUP, the TUP, 2.7 mm radial to the TUP, and 5.4 mm radial to the TUP. For each of the five locations on the TCL, the median of the SWV measurements obtained from the ten trials was used as a representative value for each subject. The median instead of the mean value was used for analysis because there were outliers with SWV measurements greater than 10 m/s, which were displayed as “HIGH” instead of numerical values. Another two-way (2×3) repeated measures ANOVA was used to examine the dependence of the SWV measurements on compression and tissue type. The first independent variable, compression, had two levels: without compression and with compression. The second independent variable, tissue, had three levels: the TCL, the skin, and the thenar muscles. For each tissue type, all 50 SWV measurements made at the five locations during the ten trials were combined and the median was used to represent the whole tissue, accounting for spatial heterogeneity. Post-hoc Tukey tests were performed for all pairwise comparisons. The TCL to skin SWV ratio and the TCL to muscle SWV ratio were also calculated. Paired t-tests were used to investigate the effects of compression on these ratios. All statistical analyses were performed using SigmaStat 3.5 (Systat Software, Inc., San Jose, CA, USA). Tests were considered significant if the p-value was below 0.05.

## Results

**Figure 3:**
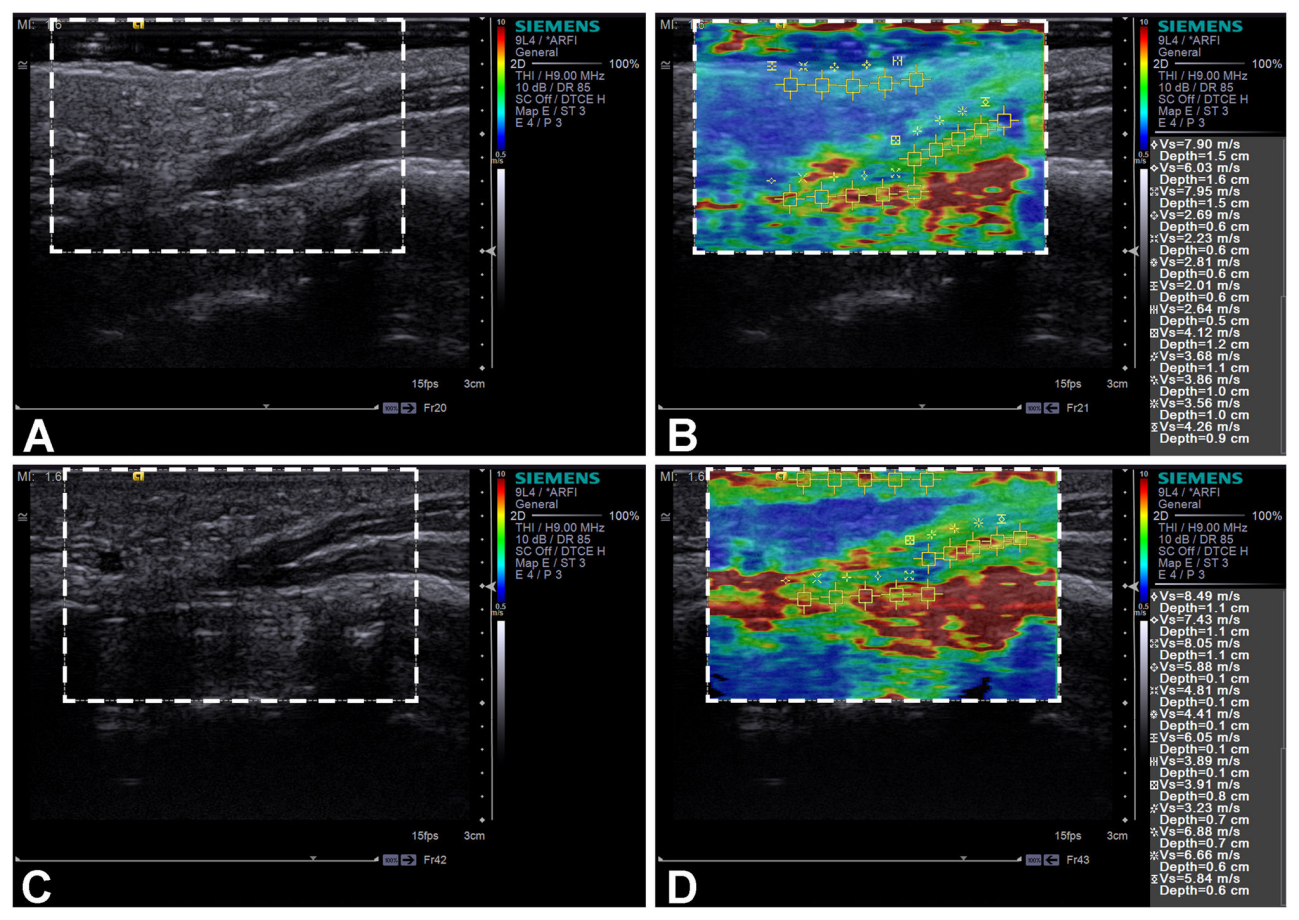
Representative B-mode and ARFI ultrasound images of the transverse carpal ligament. For the session without compression, a thick ultrasound gel layer is visible in both the B-mode (A) and the ARFI (B) ultrasound images. For the session with compression, the skin fold on the palm is flattened and the gel layer is visually absent in both the B-mode (C) and the ARFI (D) ultrasound images. SWV color maps of 30 mm (width) × 20 mm (depth) are shown in (B) and (D). The display scale of the color map is 0.5-10 m/s. The color correlates with tissue stiffness. In general, red, green, and blue areas correspond to high, medium, and low stiffness regions, respectively. Fifteen ROIs were placed on each of the SWV color map to obtain the shear wave velocity measurements.

For the session without compression, a thick ultrasound gel layer is clearly visible at the top of both the B-mode (Figure 3A) and the ARFI (Figure 3B) ultrasound images. The SWV color map (Figure 3B) provides visualization of quantitative stiffness differences between the TCL and the surrounding tissues. The TCL appears to be heterogeneous and contains regions of red and green, corresponding to high and medium stiffness regions. The skin is more spatially homogeneous with a blue hue, corresponding to low stiffness. The thenar muscles also appear to be relatively homogeneous with green and blue hues. The regions near the carpal bones (i.e. trapezium and hamate) appear to be red, corresponding to high stiffness, while the other soft tissues (e.g. fat) appear to be blue, corresponding to low stiffness. Note that there is some artifact (i.e. red regions with high stiffness) in the top portion of the SWV color map. This artifact is likely due to boundary effects at the surface of the ultrasound transducer. It may be difficult to focus ultrasound beams at such shallow depths for generating ARFI excitation beams and accurately tracking shear wave propagation. This can lead to the artificially higher SWV measurements in the very top portion of the SWV color map. For the session with compression, the skin fold on the palm is flattened and the gel layer is visually absent in both the B-mode (Figure 3C) and the ARFI (Figure 3D) ultrasound images. All tissues in the SWV color map captured with compression (Figure 3D) grossly appear to have hues corresponding to greater stiffness when compared to those without compression (Figure 3B).

A total of 4,500 SWV measurements were obtained in this study (15 subjects × 2 sessions/subject × 10 trials/session × 15 measurements/trial). No “NA” measurements were observed, indicating that all SWV measurements were made within regions of good shear wave signal to noise ratio. There were 16 “HIGH” measurements, suggesting that occasionally the tissue stiffness was beyond the capability of the current ARFI software and hardware. These “HIGH” measurements were treated as 10 m/s in further data analysis and should not affect the results since the median instead of the mean was used to represent each subject.

**Figure 4:**
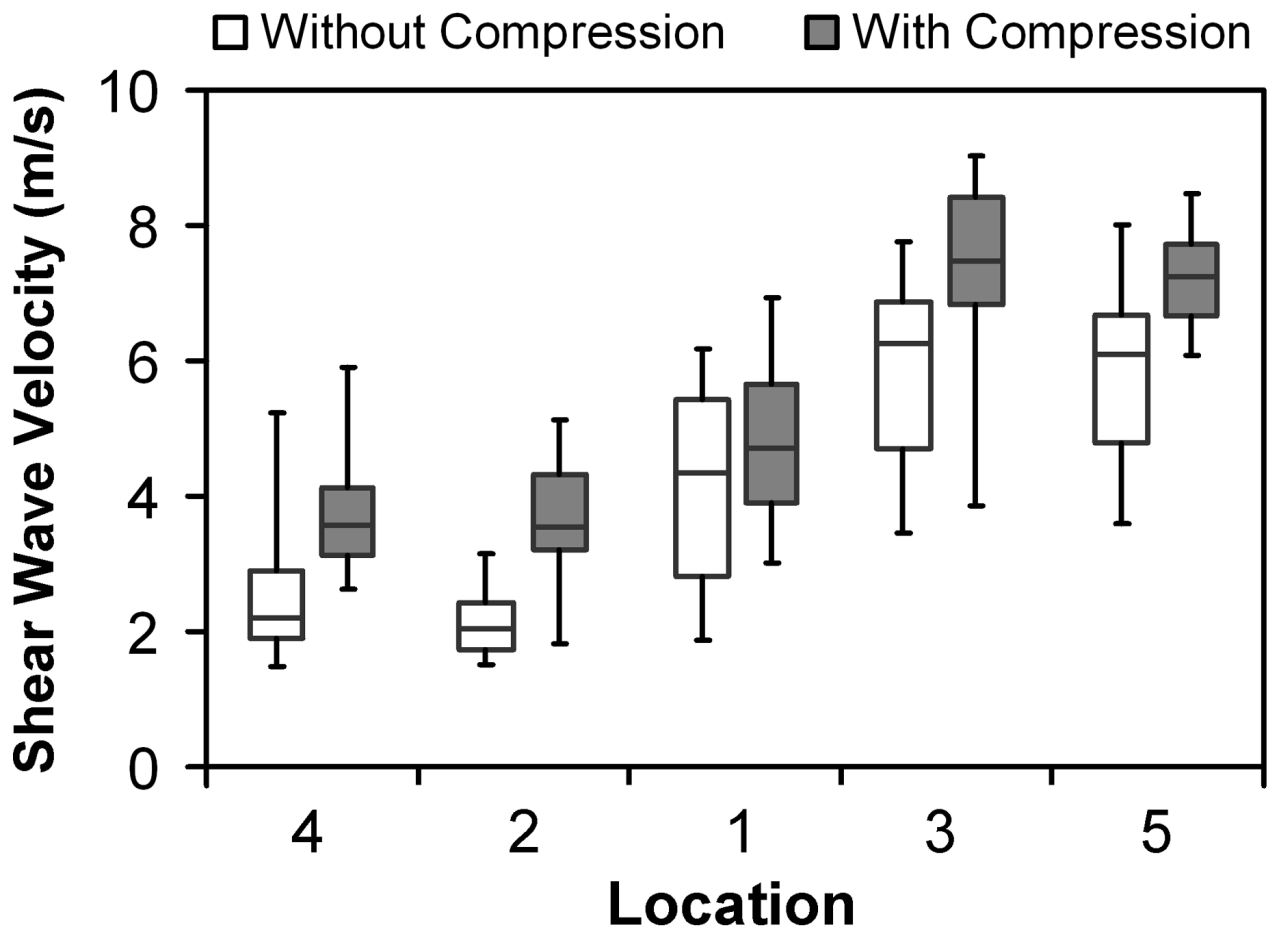
Shear wave velocity of the transverse carpal ligament by measurement location for one representative subject.

**Figure 5:**
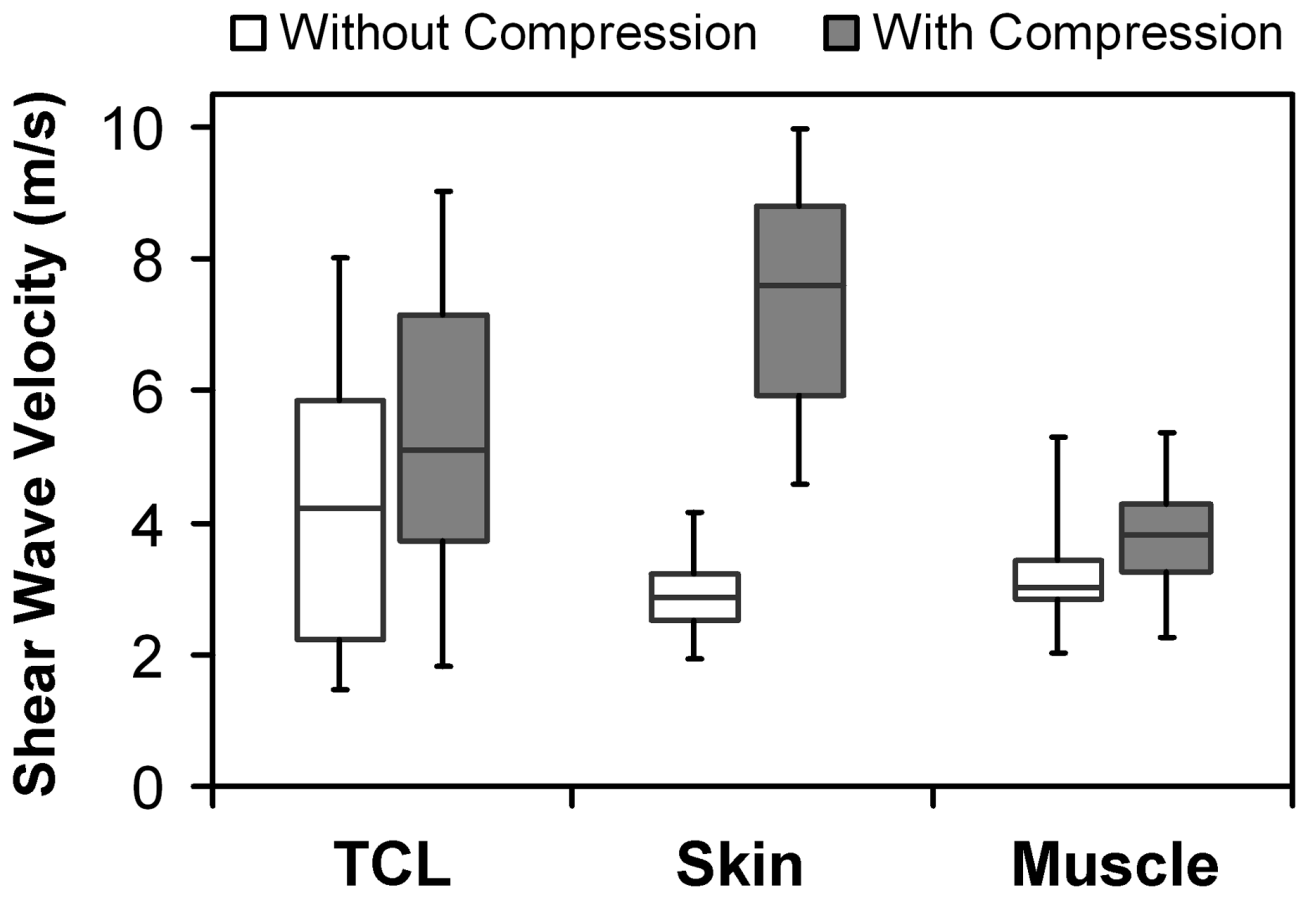
Shear wave velocity of the soft tissues by tissue type for one representative subject.

Box and whisker plots of the SWV measurements collected across all trials at the five locations on the TCL of one representative subject are shown in Figure 4. Large data scattering in the SWV measurements was observed at all five locations for both the session without compression and the session with compression. Therefore, it was essential to collect a relatively large number of trials and to use the median to represent each subject. Box and whisker plots of all the SWV measurements obtained from all trials on the three tissues from one representative subject are shown in Figure 5. For the session without compression, the SWV values obtained from the TCL showed large variability, while those measured from the skin and the thenar muscles had relatively small variability. For the session with compression, the SWV measurements of the skin had a dramatically greater variability than those without compression, whereas the SWV measurements of the TCL and the thenar muscles had variability similar to those without compression. This suggests that compression had a greater effect on the most superficial tissue, the skin, than on the TCL and the thenar muscles.

**Figure 6:**
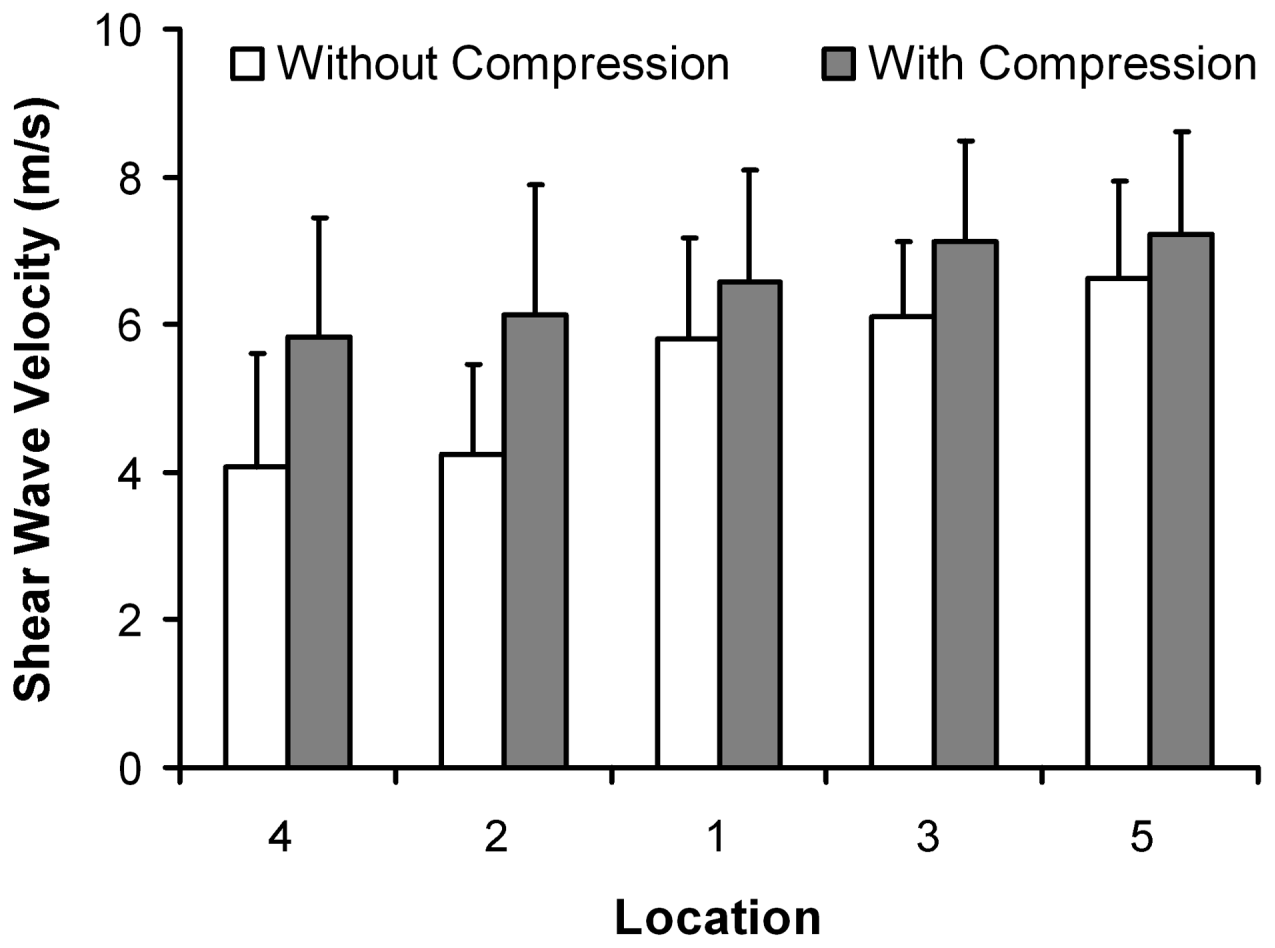
Shear wave velocity of the transverse carpal ligament by measurement location for all fifteen subjects. Mean and standard deviation are shown.

Two-way repeated measures ANOVA revealed that the SWV measurements of the TCL were significantly dependent on compression (p < 0.001), measurement location (p < 0.001), and the compression × location interaction (p = 0.011). The SWV measurements of the TCL without compression were significantly smaller than those with compression (Figure 6). Pairwise comparisons demonstrated that the SWV measurements made within the muscle-attached region of the TCL (Location 1, 3, and 5) were significantly larger than those made outside of the muscle-attached region (Location 2 and 4) (p < 0.05), while there were no differences among the locations in each region (p > 0.184 for Location 1, 3, and 5; p = 0.948 for Location 2 and 4). For the session without compression, the average SWV values of the TCL were 5.80-6.63 m/s within the muscle-attached region and 4.06-4.24 m/s outside of the muscle-attached region. For the session with compression, the average SWV values of the TCL increased to 6.59-7.23 m/s and 5.83-6.13 m/s within and outside of the muscle-attached region, respectively.

**Figure 7:**
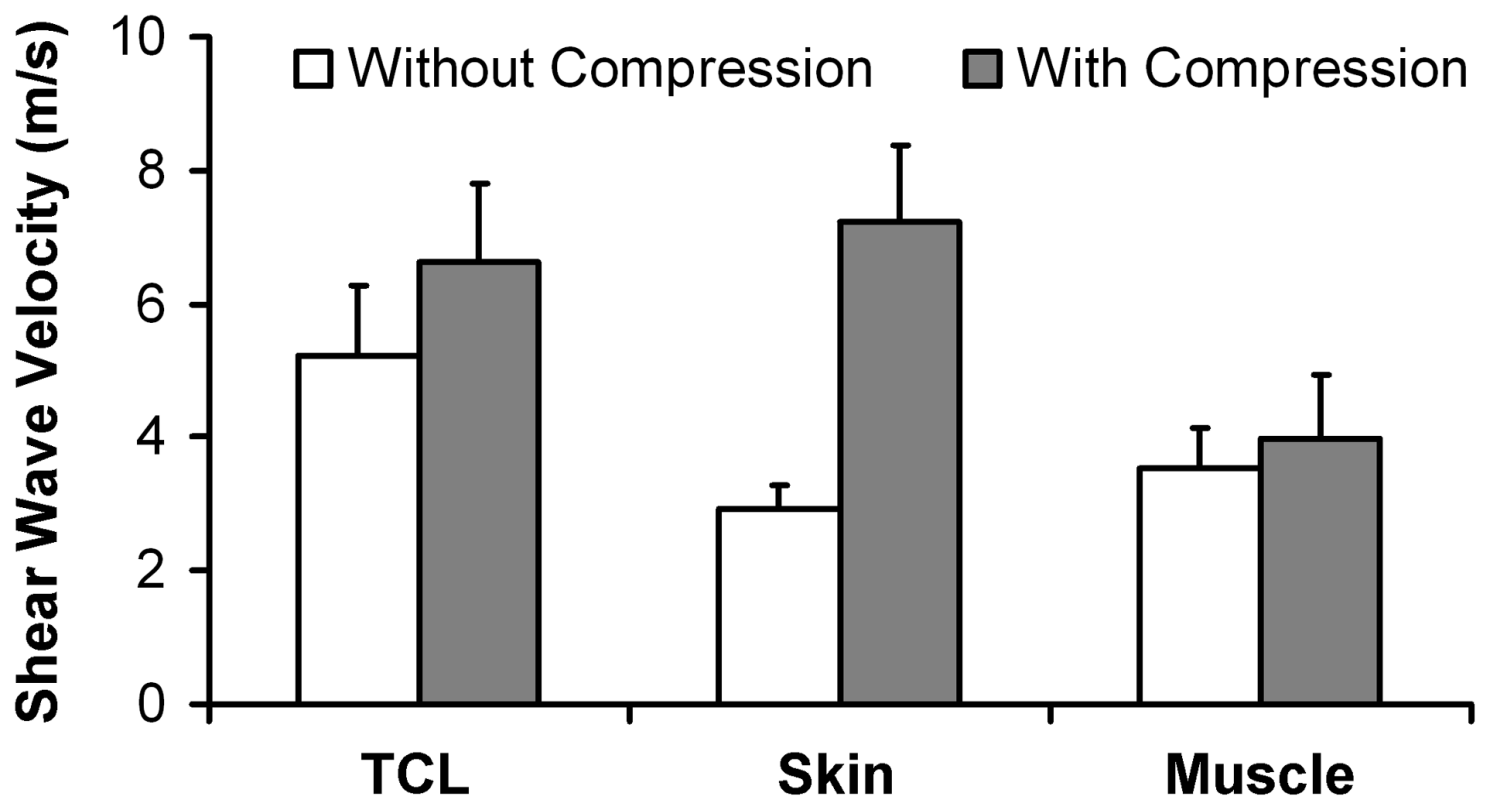
Shear wave velocity of the soft tissues by tissue type for all fifteen subjects. Mean and standard deviation are shown.

Statistical results also indicated that there were significant main effects of compression (p < 0.001) and tissue type (p < 0.001), and a significant compression × tissue interaction effect (p < 0.001) on the SWV measurements. The SWV measurements of the TCL, the skin, and the thenar muscles made without compression were smaller than those made with compression (Figure 7). The SWV of the TCL changed from 5.21 ± 1.08 m/s without compression to 6.62 ± 1.18 m/s with compression, a significant increase of 27% (p < 0.001). The SWV values of the skin were 2.92 ± 0.37 m/s and 7.24 ± 1.14 m/s for without compression and with compression, respectively, demonstrating a dramatic increase of 148% (p < 0.001) under compression. The SWV of the thenar muscles increased from 3.53 ± 0.61 m/s without compression to 3.97 ± 0.96 m/s with compression, but this change was not significant (p = 0.09). Furthermore, the TCL to skin SWV ratio significantly changed from 1.82 ± 0.49 without compression to 0.93 ± 0.22 with compression (p < 0.001). The TCL to muscle SWV ratio also significantly changed from 1.50 ± 0.31 without compression to 1.71 ± 0.34 with compression (p = 0.042).

## Discussion

Previously, the mechanical properties of the TCL have only been studied in vitro using cadaveric specimens [8,12,13]. In this study, the in vivo stiffness of the TCL was investigated by measuring the SWV using ARFI imaging. The variability of SWV measurements and the effects of measurement location and ultrasound transducer compression on the SWV measurements were also explored.

For the session without compression, the TCL SWV measurements showed greater variability compared to the skin and the thenar muscles. Three factors may contribute to this. First, the TCL SWV values were found to be dependent on the measurement location. Second, the average TCL thickness (~1.5 mm) [30] is slightly smaller than the depth of the ROI box (2 mm). Therefore, the TCL SWV measured within the 2 mm × 2 mm ROI box may be biased due to the inclusion of surrounding soft tissues (e.g. fat and muscles). Third, the TCL is anisotropic so slight changes in the orientation of the ultrasound transducer may cause variation in the TCL SWV measurement. Nonetheless, ARFI imaging is valuable in clinical applications as it can noninvasively quantify the TCL stiffness in vivo.

For the session with compression, the variability in the SWV measurements of the TCL and the thenar muscles was similar to that without compression, while the skin SWV variability increased dramatically. Two sources of variation and bias may cause the increased variability in the skin SWV under compression. First, the transducer compression was not quantitatively controlled and the variation in compression may affect the skin more than the TCL and the thenar muscles as the skin is the most superficial tissue. Second, artifacts at shallow depths close to the transducer in ARFI imaging may contribute to the increased skin SWV variability. Therefore, extra caution is needed when interpreting the SWV measurements obtained on superficial tissues by ARFI imaging.

We found that the SWV measurements of the TCL were dependent on the tissue location. The SWV values of the TCL within the muscle-attached region (i.e. the TUP, 2.7 mm, and 5.4 mm radial to the TUP) were greater than those outside of the muscle-attached region (i.e. 2.7 mm and 5.4 mm ulnar to the TUP). This finding suggests that the muscle-attached portion of the TCL is stiffer than the non-muscle-attached portion. This agrees with a previous cadaveric study using biaxial tensile testing [12] which showed that the radial segments of the TCL had greater moduli than the ulnar segments. The difference in the TCL stiffness at various tissue locations may be explained by tissue adaptation under biomechanical interaction between the TCL and the thenar muscles. A previous study [7] showed that the TCL was pulled volarly during thenar muscle contractions. This repetitive, mechanical stimulation to the TCL may cause tissue remodeling and then lead to a thickened and stiffened TCL. However, Main et al. [13] showed that the average TCL stiffness within the muscle-attached region was lower than that outside of the muscle-attached region based on volar/dorsal indentation tests. Since the TCL is known to be anisotropic, its mechanical properties may differ in the ulnar/radial ( [12] and the present study) and volar/dorsal [13] directions.

The SWV measurements of the soft tissues quantified in this study were found to be dependent on the compression from the ultrasound transducer. Similar effects of compression on the SWV measurements were previously demonstrated on breast [27], kidney [28], muscle and tendon [23] tissues. In addition, we found that the effect of the compression varied by tissue type. The skin, as the most superficial tissue, was affected most by the compression, with a dramatic increase of 148% in the SWV. Note that the SWV measurements were found to be dependent on the imaging depth, with greater SWV values measured at smaller imaging depths [31]. Since the skin was closer to the transducer in the session with compression than without compression, the bias due to imaging depth might also contribute to the higher SWV of the skin under compression. The SWV of TCL only increased 27%, while the SWV of the thenar muscles did not significantly change with compression. Therefore, the skin and the thenar muscles were not found to be good reference tissues by which to normalize the SWV of the TCL and to eliminate the effects of compression. Since compression from the transducer significantly affects the tissue stiffness and can lead to variations in the SWV measurements, care must be taken to minimize the soft tissue compression from the ultrasound transducer. We would recommend the operator to apply and maintain a thick layer of ultrasound gel so the compression from the transducer is negligible during ARFI imaging.

This study had a few limitations. First, only male subjects were recruited for this study. It is likely that the same experimental protocol can be used on females given the similar hand anatomy for both sexes. Second, only the distal level of the carpal tunnel was investigated. The distal level was chosen because the median nerve is most compressed at this level as shown in previous studies [32,33]. Third, the maximum SWV value that the current ARFI software and hardware can measure is 10 m/s. Some SWV measurements of the soft tissues were greater than 10 m/s and were displayed as “HIGH” instead of a quantitative value. This technical limitation may be addressed with improvements of the ARFI algorithm and the ultrasound transducer. Fourth, the 2 mm × 2 mm ROI box might have included some soft tissues surrounding the TCL as the thickness of the TCL was shown to be, on average, 1.5 mm [30]. The SWV for the TCL measured within the 2 mm × 2 mm ROI box may be slightly biased due to the inclusion of other softer tissues adjacent to the TCL. Fifth, effects of other material properties, such as anisotropy and viscosity, on the SWV measurements were not investigated. Sixth, only one observer performed one session for each compression condition. More investigations are needed to establish intra- and inter-observer reliability of the SWV measurements.

ARFI imaging is a valuable tool that can be used to noninvasively assess the in vivo stiffness of the TCL and has the potential to identify pathomechanical changes of the tissue associated with carpal tunnel syndrome. As the SWV measurements were dependent on tissue compression, it is recommended to standardize the ARFI imaging procedure by maintaining a visible layer of ultrasound gel to eliminate mechanical interference at the transducer-tissue interface.
